# On Feticide

**Published:** 1820-02

**Authors:** W. Hutchinson

**Affiliations:** Sackville-street


### Original Communications, Select Observations, etc.

### MEDICAL JURISPRUDENCE.

#### Dissertation I.—*On Feticide.*

§. II. *On the Moral, Political, and Legal, relations of Feticide.*

TO treat in a comprehensive manner of the moral and political relations of feticide, would require such an extensive dissertation on the nature and influence of the human passions, and the policy of civilized societies, as would be incompatible with the limits to which these articles must be here confined. A view of their principal traits is all that can here be given.

The existing laws of England attribute the same penalty to the crime of *feticide** as to that of infanticide, although the former is in itself of a different and much less heinous nature. A little reflection will render this evident. It is an indisputable fact, that the affections of men are chiefly, and in the state of savage life almost solely, excited by the impressions the objects of them make on their senses. Now, the fœtus in the womb has never made such impressions on the mother; and hence it is often found, that a woman of the best moral disposition has but little tender affection for her infant until she has contemplated it for some time, or until she has been sucked by it. Men, generally, (except when circumstances relating to social life influence their passions) hear of the fate of their newly-born progeny with great indifference, and but rarely experience much affection for them, until some degree of moral intercourse has become established. These circumstances are sufficient to indicate that the destruction of a fœtus in the womb does not arise from so great an aberration from the natural sentiments as the murder of an infant. The well-known remark of one of our greatest philosophers favours the same opinion; he said, if men flew about in the air, they would be destroyed by other men, when they were at so great a distance as to appear no larger than a bee, with as little remorse as we shoot a bird, provided it were done unintentionally: so much do our sentiments depend on sensual impressions. But feticide is an act that is not committed without some degree of reasoning on its object and con-

* This term is, of course, used to signify *wilful* destruction of the fœtus. It may be right to remark here too, that the subject is considered in the view of *feticide*, not in that of abortion, which is merely one means of producing *feticide*. The laws, on the contrary, consider it in the view of abortion only. This can cause no confusion to the medical reader, as the same laws are equally applicable to all the varieties of feticide. The writer has, however, chosen to treat of the subject in the proper physiological manner. Involuntary feticide will be treated of under the head of *homicide*.

sequences; and this must be influenced by various circumstances, peculiar to different states of civilized life and existing moral institutes. Whilst some of the reflections that arise in a woman meditating the commission of the crime, excite her to it with all the eagerness that the dread of the infamy we attach to want of chastity can produce; others accompany them, which lead her to derogate from its guiltiness. She calculates the danger a fœtus undergoes in the act of parturition; and, consequently, judges that its destruction is not so great a social evil as the suppression of the life of an infant.

Whether or not this notion be correct, and how far, in this case, established moral and legal institutes should take the place of natural sentiments and the most obvious reasonings, in persons educated in civilized societies, are questions of the highest importance: but this is not a proper place for their discussion. Yet, the view here given of the subject would be too far imperfect without something like a general account of the politics of those nations respecting it, whose manners and laws, have had some influence on the formation of those of England, as a mean of determining the relation they bear to the commission of this crime.

In several of the ancient nations of Europe, feticide was tolerated as a private act, and indirectly favoured by the laws relating to the preservation of infants; the destruction of whom, in several states, though on a superficial view, apparently dictated by the grossest barbarism and wanton cruelty, will be found, on deeper investigation, to have arisen from circumstances rendering it almost necessary; that is, it was so in connection with other political institutes. It was adopted almost solely in poor republican states, living by the spoils of war, and neglecting agriculture. At Sparta, it was unavoidable during the existence of the other laws of LYCURGUS, in order to preserve the number of citizens equal to that of the lots of land; and the evil was there rendered subservient to the preservation of the martial power of the people, by consigning the most weak and deformed infants to the *Barathron*.

A similar point of policy was adopted by the people of Cathea. At the age of two months, infants were presented before certain magistrates, who selected those which were to be destroyed. The Celts endeavoured to lessen the apparent cruelty of the same practice, by making it a sort of religious ceremony. They laid newly-born infants on a shield, and placed them thus on the stream of a river; those who arrived safe at a certain point were preserved, whilst those who were drowned, they considered perished by the will of the Fates, not by the hands of men.

No nation can have committed a crime so contrary to the ordination of nature from mere cruelty or savage barbarity. The cause may not be known to us, but some political reason, the existence of which may now have ceased, without doubt, originally gave rise to it, and it has been perpetuated by custom.* Travellers state, that the production of abortion and infanticide, are commonas private crimes in many savage nations of America. The reply of the woman of a nation bordering on the Oroonoko † to father GUMILLA, who expostulated with her on the enormity of her action, will show, that it is there committed from reasons analogous to those leading to it in Europe at the present period ; except that here the woman effects it to avoid infamy ; there, to save her progeny from misery. But, in no other nation does feticide appear to have been so common an act as it was in Rome, at the time of the Cæsars. The crime here arose, in a great measure, from the doctrine of the *Stoics*, who regarded the fœtus, before its birth, merely as a portion of the viscera of the mother ;‡ not as a creature having individual existence, much less as a human being : and these notions regulated the political laws.§ The infant was not considered a member of society until it had been acknowledged by its parents, and applied to the breast of the mother ; and the murder of it was only punished when such an act was productive of prejudice to some one of its family, &c.

These notions and laws, and the manners consequent on the moral corruption arising from inordinate luxury, made feticide

---

* This is the case, probably, with respect to the people of the Isle Formosa. Women there are not allowed to bring forth living children before the age of thirty-five, and abortion is produced by a *priestess:* a custom which, probably, when instituted, was the only means of saving the nation from perishing by famine, from over-population. Such points of policy have been connected with religious institutes by shrewd men, as the best way to secure their fulfilment ; and generally when this has been effected, they descend through many series of generations.

† "I wish to God, father, that my mother had, by my death, prevented the manifold distresses I have endured, and have yet to endure as long as I live. Had she kindly stifled me at my birth, I should not have felt the pain of death, nor numberless other pains to which life hath subjected me. Consider, father, our deplorable condition. Our husbands go to hunt with their bows and arrows, and trouble themselves no further; we are dragged along, with one infant at our breast and another in a basket. They return in the evening without any burden ; we return with the burden of our children ; and, though tired out with a long march, are not permitted to sleep, but must labour the whole night in grinding maize to make chica for them. They get drunk, and, in their drunkenness, beat us, draw us by the hair of the head, and tread us under foot. What kindness can we show to our female children, equal to that of relieving them from such servitude? I say, would to God that my mother had put me under ground the moment I was born!"

‡ *Pars ventris.* See the law De inspiciende ventre. I. § 1.

§ Plato said, an infant in the uterus was animated, because it moved as well as vegetated ; but the stoics considered it to be a part of the belly of the mother, and not an animal ; and as the fruits of trees, when ripe, separate from their parent stock, so do fœtuses come from the womb at the epoch of their maturity. PLUTARCH, de placit. philos. lib. V. § 15.

so general a practice, that Juvenal says, hardly any lady of consequence, living in a fashionable manner, produced living children.*

But, about the time of ANTONINUS, when the opinions of the ACADEMICS became prevalent, which taught that the fœtus became animated at a certain epoch of utero-gestation, feticide became considered a crime, though a difference was made between whether it was committed before or after it became *animated:* the former was punished by an arbitrary penalty, the latter with death.† CONSTANTINE took means to prevent the commission of it, by ordering throughout the whole of the cities of Italy, and those of Africa under his dominion, that those parents who declared themselves unable to support children, should be assisted by the state:‡ and the council held at Constantinople in 692, enacted, that the procuring abortion at any time should be punished as homicide.

The canon law in the earlier ages of Christianity, however, was much less severe than that of the later Roman emperors: it did not regard it as homicide until the fœtus was fully formed.§ The council of Elvira merely excluded for ever from participation of the sacraments, the mother who was convicted of having willfully produced abortion. The council of Ancyra in 314, and that of Lepida in 524, decreed the one a penitence of ten years, the other of seven years, and an interdiction from the sacraments during the same periods. More severe measures were, however, at length enacted ; for Pope SEXTUS the Fifth, by a bull dated the 16th of November 1588, and Gregory the Fourteenth, by one of July the 9th 1591, attributed a capital penalty to this crime. But, it is remarkable that the accomplices escaped so easily, that the former Pontiff only pronounced eternal irregularity against priests, and excommunication of laics, who had been abettors of it.

"In England," HAWKINS says,‖ "it was anciently holden that the causing of abortion, by giving a potion to, or striking, a woman big with child, was murder. But at this day it is said to be a great misprision only, and not murder, unless the child be born alive and die thereof, in which case it seems certainly to be murder, notwithstanding some opinions to the contrary." It is HALE to whom he here alludes, who says, "an infant in its mother's womb, not being in *rerum natura*,

* Sed jacet aurato vix ulla puerpera lecto :
Tantum artes hujus (veneficæ), tantum medicamina possunt,
Quæ steriles facit, atque homines in ventre necandos.
*Sat.* vi. 495.

† See the law *Si quis necandi*, and the glossary on that of *Divus*.

‡ *Codex Theodos.* Lib. ix. tit. 15.

§ ZACCHIAS *Questiones Medico-legales*, Lib. ix. t. 1.

‖ *On the Pleas of the Crown*, I. 188. § 16.

is not considered as a person who can be killed within the description of murder; and therefore if a woman, being quick or great with child, takes any potion, or if a person shakes her, whereby the child within her is killed, it is not murder or manslaughter."*

An attempt, however, to effect the destruction of such an infant, though unsuccessful, appears to have been treated as a misdemeanor at common law;† and a statute has lately been passed, by which certain acts, intended to procure the miscarriage of a woman with child, are made highly penal.‡ After reciting that certain heinous offences, with intent to procure the miscarriage of women, had of late been frequently committed, and that no adequate means had been provided for their prevention and punishment, this statute enacts, that if any person shall, either in England or Ireland, "wilfully, maliciously, and unlawfully administer to, or cause to be administered to, or taken, by any of his Majesty's subjects, any deadly poison or other noxious and destructive substance or thing, with intent such of his Majesty's subject or subjects thereby to murder, or thereby to cause and procure the miscarriage of any woman then being *quick*§ with child," the person so offending, their counsellors, aiders, and abettors, knowing of and privy to such offence, shall be felons, and shall suffer death as in cases of felony, without benefit of clergy.||

The second section of the statute recites, that it might sometimes happen that poison or some other noxious substance might be given, or other means used, with intention to procure abortion, when the woman might *not be quick* with child at the time, or it might *not be proved* that she was quick with child, and enacts "that if any person or persons shall wilfully administer to, or cause to be administered to, or taken, by any woman, any medicine, drug, or any other thing whatever, or shall use or employ, or cause to be used or employed, any instrument or other means whatever, with *intent* thereby to cause or procure the miscarriage of any woman *not being* or *not proved to be* quick with child, at the time of administering such things or using such means, that then and in every such case, the person or persons so offending, their counsellors, aiders, or abettors, knowing of and privy to such offence, shall be and are hereby declared to be guilty of a felony, and

---

* HALE, I. 433.

† CHITTY, *Crim. Law*, 798, procured from the Crown office, Mich. T. 42. Geo. III.

‡ 43 Geo. III. c. 58. s. 1.

§ "Life, in the contemplation of the English law, begins as soon as a child is able to stir in its mother's womb."—BLACKSTONE's *Comment.* vol. i. p. 129.

|| It is proper to remark, that no difference is made whether the mother herself commit the crime, or another person.

shall be liable to be fined, imprisoned, set in and upon the pillory,* publicly or privately whipped, or to suffer one or more of the said punishments, or to be transported beyond the seas, for any term not exceeding fourteen years, at the discretion of the court."†

Thus the English laws have acquired increased severity, whilst those of the other nations of Europe in general have become progressively more lenient. By an edict of Henry the Second of France, the production of abortion, infanticide, and the exposition of infants, received the same punishment—that of death. This severity, however, did not long exist; for, in 1576, a girl convicted of exposure of her infant, was only condemned to be whipped and marked. But, it was not until the construction of the *penal code* of 1791, that the penalty of feticide was altered, though it was by that code punished with twenty years of imprisonment in *irons*. By this law, however, it was only the accomplices of the crime that were punished: the mother escaped. M. MARC‡ attributes the impunity which the mother enjoyed, to the difficulty of establishing the crime without her own confession; and this it was supposed could not be obtained, if she herself existed in fear of judicial penalties.

The code of the year 1810 is however more perfect, at the same time that it is more lenient to the abettors. It enacts that—

"Whoever by aliments, potions, medicines, violence of any kind, or by any other means, shall have procured the abortion of a pregnant woman, whether or not she consented to it, shall be punished by imprisonment.

"The same penalty shall be pronounced against the woman who shall herself have produced her own abortion, or who shall have consented to use means pointed out or administered to her for that purpose, if abortion shall thence ensue.

---

* The punishment of the pillory has been since taken away, by the 56 Geo. III. c. 138.

† Medical readers will remark, respecting the first section of the above statute, that, although it speaks of administering medicines, &c. to a woman *proved to be quick with child*, with the *intent* to procure abortion, as highly penal, without saying any thing respecting the effects of such medicines, it is only when such medicines *actually produce abortion* that the penalty can be attributed to the act alluded to; because, unless abortion takes place, it cannot be proved that the woman was with child at the time the medicines were used, much less that she was then *quick with child*. But, as the *intent* to produce abortion, whether or not the woman who may be the object of it be with child even, is itself a crime, it would be punishable by common law; but only as a *misdemeanor*, not as an act of *felony*. Medical practitioners will here perceive the importance of firm and decisive conduct in a Court of Justice on an enquiry of this nature. The authorities cited (page 3 of the Journal) in the last article, will be sufficient support for them in their protestations against the validity of every testimony but one, of the existence of pregnancy, as well as of the occurrence of abortion,—that is, inspection of the fœtus itself.

‡ *Dictionaire des Sciences Medicales: Art. Avortement.*

"Physicians, surgeons, and other medical practitioners, as well as pharmaceutists, who shall have indicated or administered those means, shall be condemned to forced labour for a certain period of time, in cases where abortion shall have taken place."*

The laws of Germany† punish with from two to six years imprisonment a woman (or her aiders, &c.), who, by potions or any other means, shall have wilfully produced abortion within the first thirty weeks from the time of conception; and the penalty is protracted to eight, or at the utmost to ten years, when such a crime has been committed within the last month of pregnancy.

The laws of Bavaria enact similar measures.‡

In the Italian code§ it is established, that if a woman has used means with the intent to produce abortion, and this shall *not* have taken place, she is to be punished by imprisonment for a period of from six months to one year; but, if abortion has been the consequence of such means, the imprisonment is to be of from one to five years duration. The same penalties, but with exacerbations, are enacted against the father of the fœtus if he has been an accomplice in the crime. Finally, the delinquent who, against the will of the mother, shall have caused abortion, or have made an attempt to cause her abortion, is to be punished by from one to five years severe imprisonment; and if the life of the mother has thereby been brought into danger or her health been injured, the duration of the penalty shall be from five to ten years.‖

The particular severity of the English laws respecting feticide, in relation to those of several other nations of Europe, is not a singular point in our penal code; it is conformable to many other parts of it. This method of administering justice, which applies capital punishments to many kinds of offences, but inflicts it only upon a few examples of each kind, has, however, been advocated in its general application by a great moralist,** "because (he says) by this expedient few actually suffer death, whilst the dread and danger of it hang over the crimes of many."†† And in his further support of it, he says:

---

* *Code Penal* (of 1810), § 317.

† *Allg. pr. Gesetzb.* Th. 2. tit. 20. § 985, 986.

‡ *Strafgesetzb. für das K. Bayern*, art. 172.

§ *Codice dei delitti*, c. xvii. § 129, 130, 131, 132.

‖ When this article was required for the press, the writer had not ready a precise account of the laws of Scotland respecting feticide; but the want shall be supplied in the next number of the Journal.

** Dr. Paley, in his *Principles of Moral and Political Philosophy*, vol. ii. p. 281. and *seq.* 17th edition.

†† According to the report printed under the direction of the Secretary of State for the Home Department, 528 persons were capitally convicted in London and

"Upon this plan it is enough to vindicate the lenity of the laws; that *some* instances are to be found in each class of capital crimes which require the restraint of capital punishment, and that this restraint could not be applied without subjecting the whole class to the same condemnation."

It is not intended here to discuss the propriety of these sentiments in their general application, but to shew the incorrectness of them in regard to feticide. In the greater proportion of the crimes to which Dr. Paley alludes, the jury have the power of finding a verdict for *felony*, though not for capital felony, and this is what is commonly done; and thus some degree of punishment may be conferred on every culprit. But on a charge of feticide, they must either find for the fact or acquit the delinquent; and in the application of mercy after conviction, the supreme authority must totally pardon the culprit and all the accomplices, if it be at all exerted. The consequence of this is, that charges are but very rarely brought for feticide, because those persons who should prosecute think the penalty too severe, and they are especially disposed to pity one of those who must become its victims: or else witnesses violate their oaths and conceal what would lead to conviction. That, however, feticide is so rare an action in England, can hardly be supposed; and it is a crime in which the escape of the accomplices especially,* (without whom it is in all probability hardly ever committed,) is an evil of the highest magnitude; for they are often persons with whom it is familiar, and who make the commission of it a sort of regular profession. The sole object of human punishments, it is admitted by all moralists and legislators, is the prevention of crimes, and to this end they operate principally by the terror of example; but to effect this, punishment must, as nearly as possible, be the certain consequence of committing them. According to the present practice, however, the punishment is merely the *possible*, but by no means the *probable*, consequence of the crime, and the chance of this is so very slight, that evil-disposed persons under the urgent circumstances which commonly incite them to feticide, very readily contemn it.

Were the penalty less severe, there can be no doubt that prosecutions would be more frequent, and conviction, or the infliction of penalty, not so extremely rare an occurrence as it has been in England for many years past.

---

Middlesex from the years 1802 to 1808 inclusive, of which only 67 were executed. What proportion of those instances of the infliction of penalty was for the crime of feticide, cannot be calculated, because the nature of the crime is not stated in the reports: but it must have been very small—perhaps none.

* There can be but little doubt that in cases of feticide of bastards (its general application), the father is almost always an accomplice, and the wisdom of the Italian laws, in making a distinction of punishment for him; and for endeavours to procure abortion against the will of the mother, or unknown to her, must, therefore, be highly approved.

It may be asked, would the fear of a milder punishment than that of death, have sufficient influence with regard to the prevention of the crime? One of our greatest lawyers would reply in the affirmative. In his observations on the administration of the criminal law of England, Sir SAMUEL ROMILLY says, "What the public safety requires, is that crimes should be prevented by the dread of death, whenever the dread of a less evil will not be efficacious. In no other way can the public safety require the death of any individual. Now it is universally agreed, that the certainty of punishment is much more efficacious than any severity of example for the prevention of crimes. Indeed, this is so evident, that if it were possible that punishment, as the consequence of guilt, could be reduced to an absolute certainty, a very slight penalty would be sufficient to prevent almost every species of crime, except those which arise from sudden gusts of ungovernable passion."

If this opinion be correct, it becomes an highly important question in jurisprudence, whether or not the circumstances under which feticide is committed, especially as far as the mother is concerned, do not show that it might be placed with the cases to which Sir Samuel Romilly first alludes.

W. HUTCHINSON.*

*Sackville-street, Jan.* 12*th*, 1820.

---

* W. H. had considered that these articles on Medical Jurisprudence might without impropriety be anonymous; but several persons whose judgment he respects, have expressed the opposite opinion; for, it has been remarked, though there is nothing in a name, yet when readers have on examination found the statements made to be correct, and the references to the opinions of others accurate, they will afterwards rely with confidence on what is advanced, provided they perceive the identity of the author. The method of them, however, shall not be altered;—there will be the same care taken that has been done, to support the most important opinions, when it is possible, by the most estimable authorities, especially with respect to physiological facts. The judicious reader will distinguish this conduct from pedantry, as well as from the practice of citing the names of authors and the titles of their books, without knowing any thing of their contents, from PLOUQUET's digest and the like catalogues. The chapter and page of every cited author (except occasionally, in a few instances, where it has by accident been omitted in the writer's collected notes) shall be precisely stated, that those who desire to examine their accuracy may do it without trouble, or readily refer to them for further information. These articles will be merely selections (as comprehensive as the limits of the Journal will permit) from very copious notes the writer has long been collecting for an extensive work on Medical Jurisprudence, Medical Police, &c.; and, therefore, should any reader think any points are not treated with sufficient detail, or desire future information on them, the error shall be corrected, and such information either publicly or privately supplied. The readers of the Medical and Physical Journal are also assured that, though the work above alluded to may be published before these articles extend to every subject, the promise made to continue them in the manner indicated shall not be broken.

